# Evaluation of Serum Lipids, Biochemical Parameters, Selected Antioxidant Elements and Oxidative Stress Profiles in Late Pregnant Jennies with Hyperlipemia

**DOI:** 10.3390/vetsci11120664

**Published:** 2024-12-18

**Authors:** Qingze Meng, Yang Shao, Wei Li, Jia Lu, Xinyue Wang, Liang Deng

**Affiliations:** Department of Animal Genetics, Breeding and Reproduction, College of Animal Science and Veterinary Medicine, Shenyang Agricultural University, Shenyang 110866, China; 15502462577@163.com (Q.M.); 2022220555@stu.syau.edu.cn (Y.S.); 2023220582@stu.syau.edu.cn (W.L.); 2023240732@stu.syau.edu.cn (J.L.); 2022240704@stu.syau.edu.cn (X.W.)

**Keywords:** donkey, hyperlipemia, oxidative stress, pregnancy, trace element

## Abstract

Donkeys are particularly at risk of hyperlipemia. Hyperlipemia is a metabolic disease caused by the mobilization of fatty acids from adipose tissue, which often impacts pregnant and lactating jennies (female donkeys) during periods of negative energy balance. This study aimed to evaluate the levels of lipids, biochemical parameters, selected antioxidant elements and oxidative stress parameters in late pregnant jennies affected by hyperlipemia. Compared with the healthy jennies, the hyperlipemic animals exhibited significantly elevated levels of triglycerides (TGs), total cholesterol (T-CHO) and low-density lipoprotein cholesterol (*p* < 0.05), coupled with reduced levels of high-density lipoprotein cholesterol and albumin (ALB) (*p* < 0.05). The serum levels of biochemical parameters related to liver function, such as aspartate aminotransferase, alanine aminotransferase, alkaline phosphatase (AKP) and cholinesterase (CHE), showed a significant increase in the hyperlipemia group compared to the healthy group (*p* < 0.05). The serum level of selenium was significantly lower (*p* < 0.05) and positively correlated with TGs (r = 0.85) and ALB (r = 0.73) in the hyperlipemia group. The hyperlipemic jennies showed diminished serum levels of antioxidant capacity and increased levels of malondialdehyde (MDA). The area under the curve values for T-CHO, ALB, AKP, CHE, total superoxide dismutase, glutathione and MDA were relatively high. Thus, our findings reflect metabolic disorders, liver dysfunction and oxidative stress in late pregnant hyperlipemic jennies, providing a basis for the improvement of clinical diagnostic methods and early prevention and control of hyperlipemia in jennies.

## Simple Summary

Hyperlipemia is common in donkeys and has a reported prevalence of 3–5% in the field population. In this study, the serum levels of lipids, biochemical parameters, selected antioxidant elements and oxidative stress parameters in late pregnant jennies (female donkeys) with and without hyperlipemia were evaluated and compared. Key findings showed that serum had elevated levels of lipids, including triglycerides, total cholesterol and low-density lipoprotein cholesterol in late pregnant jennies with hyperlipemia. In contrast, serum levels of selenium and antioxidant capacity diminished. This study revealed the features of oxidative stress in late pregnant hyperlipemic jennies underscored the importance of early diagnosis and intervention for hyperlipidemia in pregnant jennies. It has potential implications for improving clinical diagnostic methods and treatment strategies.

## 1. Introduction

Donkeys (*Equus africanus asinus*) have been used by humans for pack and draught work. Nowadays, the new and evolving role of donkeys in milk, meat and hide production and animal-assisted therapy is a renewed interest to human life [1]. Donkeys have a long reproductive cycle, where the gestation length lasts for a mean value of 374 days [2]. Therefore, successful pregnancies are essential for maintaining the reproductive efficiency of donkeys. However, pregnancy is a very dynamic physiological period that leads to an increase in metabolic demand [3]. In the final trimester of the pregnancy period, the jenny (female donkey) has a greater energy requirement as the fetus develops rapidly, leading to the negative energy balance in the maternal body [4].

Dyslipidemia is more frequent in donkeys than in other equids. Donkeys are particularly at risk of hyperlipemia, with a prevalence of 3–5% in the general population [5]. The higher incidence of hyperlipemia in donkeys is likely related to their efficiency to store lipids and rapid ability to mobilize fat stores [6]. Hyperlipemia is a metabolic disease caused by the mobilization of fatty acids from adipose tissue and is typically associated with periods of negative energy balance and physiologic disturbances of glucose and lipid homeostasis, which frequently affects late pregnant and lactating donkeys [7,8]. In late pregnancy, estrogen stimulates the liver to produce very low-density lipoprotein (VLDL), reducing the clearance of triglycerides (TGs) by lipoprotein lipase in the liver and adipose tissue. It leads to an increase in plasma triglyceride concentration to ensure sufficient energy substrates for normal fetal development [6]. The donkeys with hyperlipemia commonly showed clinical signs of dullness, depression, anorexia, weakness, emaciation, rough hair coat, continuous weight loss, lethargy and poor body condition [9]. They are invariably difficult to manage and mortality rates in the range of 60–80% are frequently reported, which causes significant economic losses in the global donkey industry [10].

However, hyperlipemia usually has no obvious clinical manifestations in the early stage, which may not be noticeable early on to the owners. Hyperlipemia is diagnosed by the routine measurement of serum TG concentrations in sick or at-risk donkeys [9]. Donkeys have lower LDL and cholesterol, slightly higher high-density lipoprotein (HDL), but similar VLDL concentrations compared with horses [6]. This different lipoprotein profile in donkeys could potentially contribute to their higher risk of hyperlipemia.

Oxidative damage can accelerate the pathogenic progress of hyperlipemia and its complications. In the early evolution of hyperlipidemia, oxidative stress and lipoprotein oxidation played an important role [11]. Lipids, in particular, the polyunsaturated fatty acyl chains contained in phospholipids, represent a main target of reactive oxygen species (ROS) attack [12]. LDLs represent a prominent target for an oxidative reaction [11]. An adequate antioxidant supply may help prevent the course of hyperlipemia [13]. Some trace elements and vitamins were reported to have an antioxidant capacity and be vital to maintaining normal lipid metabolism in humans and animals. The functions of selenium (Se) in the organism are mainly connected with its antioxidant properties, as it is an essential part of important antioxidant enzymes [14]. Zinc (Zn) is a component of the oxidant defense system and associated with oxidative stress in relation to its deficiency [15]. Vitamin E (VE) is the major lipophilic radical-scavenging antioxidant in vivo and protects the body from the oxidative stress mediated by active oxygen [16]. Furthermore, a meta-analysis showed that serum Se was associated with hyperlipidemia in humans [17]. VE possess anti-hyperlipidemic properties in various types of animals [18].

To date, to the best of our knowledge, little is known about the changes in the serum levels of lipids, biochemical parameters, antioxidant elements and oxidative stress parameters in jennies with and without hyperlipemia in late pregnancy; however, more detailed information on these parameters during late pregnancy in jennies is important to ensure proper diagnosis, care and disease treatment. Therefore, we hypothesized that some serum antioxidant elements might play a vital role in regulating lipid metabolism and oxidative stress in late pregnant jennies with hyperlipemia. The objective of the present study was to evaluate and compare the levels of lipids, biochemical parameters, selected antioxidant elements and oxidative stress parameters between late pregnant jennies with and without hyperlipemia.

## 2. Materials and Methods

### 2.1. Animal Welfare Statement

All experiments were approved by the Animal Care and Use Committee of Shenyang Agricultural University (Approval no. 202306017).

### 2.2. Animals

The study was conducted at a donkey breeding farm in Liaoning Province, China. Forty Chinese Liaoxi jennies in late pregnancy were included in this study. Among these jennies, twenty animals were clinically diagnosed with hyperlipemia based on the following criteria: (1) developed depression, anorexia, weight loss, a rough coat and lethargy; (2) body condition scores (BCSs) ranged from 1 to 2 (1 = poor, 2 = moderate, 3 = ideal, 4 = fat and 5 = obese); (3) serum was usually turbid and white (milky); and (4) the serum TG concentrations were over 2.8 mmol/L, according to the guidelines [9,19]. The other twenty jennies in late pregnancy were evaluated clinically following The Donkey Sanctuary recommended standards, including demeanor and behavior, body condition, temperature, pulse rate, respiratory rate, mucous membrane color, abdominal auscultation, examination of the oral cavity, ocular examination, rectal examination, peritoneal tap, ultrasound and blood examination [20]. They were confirmed to be healthy throughout the investigation. The comparison of age, parity, body weight, BCS and gestation length in hyperlipemic and healthy jennies is shown in Table 1.

Jennies were fed with dry corn stover, along with a commercial concentrate feed [21], according to the nutrient requirements stated by the National Research Council recommendations [22]. They were housed in individual boxes (3 m × 3 m) 10–15 days before the presumptive delivery until 15 days after foaling. Measures were implemented to minimize stress throughout the study.

### 2.3. Blood Sampling

Blood samples were obtained from the jugular vein between 7:30 and 8:30 a.m. to avoid alterations related to diurnal variations [23]. For the determination of serum parameters, blood samples were collected into 10 mL vacuum tubes (BD Vacutainer, NJ, USA) without anticoagulant. The blood samples were then centrifuged at 3000× *g* for 10 min at 4 °C to separate the serum. Subsequently, the serum samples were stored at −80 °C until further analysis.

### 2.4. Serum Parameter Analysis

Serum levels of TGs, total cholesterol (T-CHO), high-density lipoprotein cholesterol (HDL-C), low-density lipoprotein cholesterol (LDL-C), total protein (TP), albumin (ALB), aspartate aminotransferase (AST), alanine aminotransferase (ALT), alkaline phosphatase (AKP), cholinesterase (CHE), total antioxidant capacity (T-AOC), glutathione (GSH), total superoxide dismutase (T-SOD) and malondialdehyde (MDA) were measured using a microplate reader (GENios Plus, Tecan, Männedorf, Switzerland) following standard methods with commercially available kits (Nanjing Jiancheng Bioengineering Institute, Nanjing, China). The serum levels of Se and Zn were determined by the established procedures of atomic fluorescence spectrometry (HGF-V, Beijing Haiguang Instrument Co., Ltd., Beijing, China) and atomic absorption spectrometry (model Z-2000, Hitachi, Tokyo, Japan), respectively. High-performance liquid chromatography was used to determine the content of VE in the serum by Agilent High-Performance Liquid Chromatograph 1260 Series (Agilent Technologies, Santa Clara, CA, USA).

### 2.5. Statistical Analysis

Data were analyzed with the statistical software program SPSS for Windows, version 22.0 (SPSS Inc., Chicago, IL, USA). Data were assessed for normality by the Shapiro–Wilk test, which showed that the variables were normally distributed. Simple descriptive measures, such as mean, standard error (SE) and confidence interval (CI) values, of all parameters were estimated. According to the Youden index, receiver operating characteristic (ROC) curve analysis was conducted to estimate optimal values of cutoff, as well as to maximize sensitivity and specificity. An independent-sample Student t test was used to determine the statistical significance of differences in serum parameters between hyperlipemic and healthy jennies. Pearson correlation was performed on the serum parameters of hyperlipemic and healthy jennies. All values were presented as mean ± SE. Significant differences were considered in terms of the associated *p*-value relative to *p* < 0.05 and *p* < 0.01.

## 3. Results

### 3.1. Analysis of Serum Lipids

The serum levels of TGs, T-CHO and LDL-C showed a significant increase in the hyperlipemia group compared to the healthy group (*p* < 0.05). The serum level of HDL-C was significant lower in the hyperlipemia group than that in the healthy group (*p* = 0.002) (Table 2).

**Figure 1:**
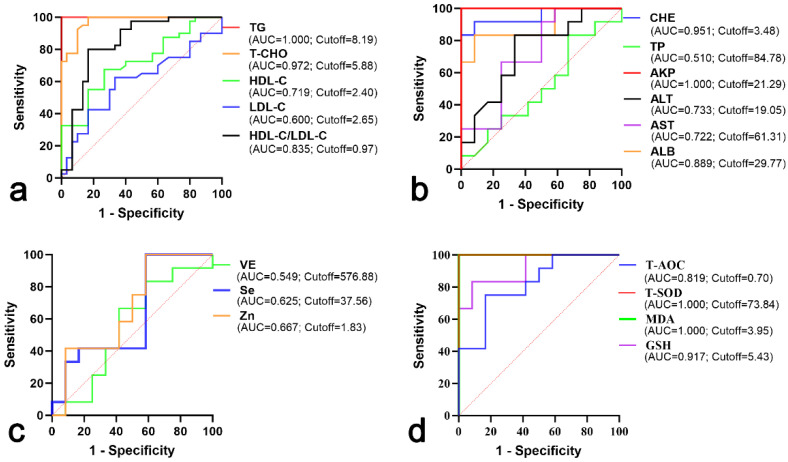
ROC of serum parameters in hyperlipemic and healthy jennies. (**a**) ROC of serum lipids; (**b**) ROC of serum biochemical parameters related to liver function; (**c**) ROC of serum selenium, zinc and vitamin E; (**d**) ROC of serum oxidative stress parameters. Abbreviations: ROC, receiver operating characteristic; TG, triglyceride; T-CHO, total cholesterol; HDL-C, high-density lipoprotein cholesterol; LDL-C, low-density lipoprotein cholesterol; TP, total protein; ALB, albumin; AST, aspartate aminotransferase; ALT, alanine aminotransferase; AKP, alkaline phosphatase; CHE, cholinesterase; T-AOC, total antioxidant capacity; T-SOD, total superoxide dismutase; MDA, malondialdehyde; GSH, glutathione; VE, vitamin E; Se, selenium; Zn, zinc.

Analytic results of the ROC curve are shown in Figure 1. The area under the curve (AUC) values of serum TG and T-CHO levels in the hyperlipemia and healthy groups were 1.000 and 0.972, with the optimum cutoff point calculated on the basis of maximum sensitivity and specificity being 8.19 mmol/L and 5.88 mmol/L, respectively. The AUCs of serum HDL-C and LDL-C were 0.719 and 0.600, with the optimum cutoff point being 2.40 mmol/L and 2.65 mmol/L, respectively. The AUC of HDL-C/LDL-C was 0.835, with the optimum cutoff point being 0.97 (Figure 1a).

### 3.2. Analysis of Serum Biochemical Parameters Related to Liver Function

The descriptive statistics and differences in serum levels of biochemical parameters related to liver function between the hyperlipemic and healthy jennies are summarized in Table 3. The serum level of ALB in the hyperlipemic jennies was significantly lower than in the healthy animals (*p* = 0.001), and the TP level was not found to be statistically different between the two groups (*p* = 0.490). The serum levels of AST, ALT, AKP and CHE showed a significant increase in the hyperlipemia group compared to the healthy group (*p* < 0.05).

The AUCs of serum TP and ALB levels in the hyperlipemia and healthy groups were 0.510 and 0.889, with the optimum cutoff points being 84.78 g/L and 29.77 g/L, respectively. The AUCs of serum levels of AST, ALT, AKP and CHE were 0.722, 0.733, 1.000 and 0.951, with the optimum cutoff points being 61.31 U/L, 19.05 U/L, 21.29 King unit/100 mL and 3.48 U/mL, respectively (Figure 1b).

### 3.3. Analysis of Serum Selenium, Zinc and Vitamin E

As shown in Table 4, the serum level of Se is lower in the hyperlipemia group compared with the healthy group (*p* = 0.031). There was no significant difference in serum levels of Zn and VE between the two groups (*p* > 0.05).

The AUC of serum Se in the hyperlipemia and healthy groups was 0.625, with the optimum cutoff point being 37.56 μg/L. The AUCs of serum Zn and VE were 0.667 and 0.549, with the optimum cutoff points being 1.83 mg/L and 576.88 nmol/L, respectively (Figure 1c).

### 3.4. Analysis of Serum Oxidative Stress Parameters

Comparisons of the serum levels of oxidative stress parameters in the hyperlipemia and healthy groups are shown in Table 5. The serum level of MDA in the hyperlipemia group was higher than that of the healthy group (*p* = 0.000), and the levels of T-AOC, GSH and T-SOD were lower than those in healthy group (*p* < 0.05).

The AUCs of serum T-AOC and GSH concentrations in the hyperlipemia and healthy groups were 0.819 and 0.917, with the optimum cutoff points being 0.70 mmol/L and 5.43 μmol/L, respectively. The AUCs of serum T-SOD and MDA were both 1.000, with the optimum cutoff points being 73.84 U/mL and 3.95 nmol/mL, respectively (Figure 1d).

### 3.5. Analysis of Correlation Among Serum Parameters

**Figure 2:**
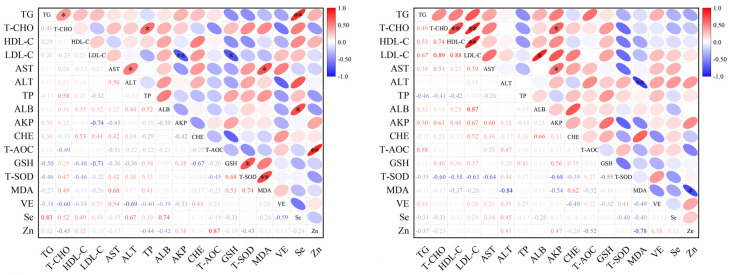
Correlation heat map of serum lipids, biochemical parameters, selected antioxidant elements and oxidative stress parameters of hyperlipemic and healthy jennies. *, *p* < 0.05; **, *p* < 0.01. Abbreviations: TG, triglyceride; T-CHO, total cholesterol; HDL-C, high-density lipoprotein cholesterol; LDL-C, low-density lipoprotein cholesterol; AST, aspartate aminotransferase; ALT, alanine aminotransferase; TP, total protein; ALB, albumin; AKP, alkaline phosphatase; CHE, cholinesterase; T-AOC, total antioxidant capacity; GSH, glutathione; T-SOD, total superoxide dismutase; MDA, malondialdehyde; VE, vitamin E; Se, selenium; Zn, zinc.

The correlations among serum lipids, biochemical parameters, selected antioxidant elements and oxidative stress parameters of hyperlipemic and healthy jennies are shown in Figure 2. The serum level of Se was positively associated with TGs (r = 0.85, *p* = 0.005) and ALB (r = 0.73, *p* = 0.014) in the hyperlipemia group. The serum level of LDL-C was negatively associated with GSH (r = 0.71, *p* = 0.033) and AKP (r = 0.74, *p* = 0.009). The serum level of Zn was positively associated with T-AOC (r = 0.87, *p* = 0.005) (Figure 2). In healthy jennies, the serum level of T-CHO was positively associated with HDL-C (r = 0.74, *p* = 0.006), LDL-C (r = 0.89, *p* = 0.008) and AKP (r = 0.61, *p* = 0.037). MDA was negatively associated with ALT (r = 0.84, *p* = 0.004) and Zn (r = 0.78, *p* = 0.038) (Figure 2).

## 4. Discussion

Late pregnancy is the most challenging and critical period of breeding jennies. The digestive tract capacity decreases as the pregnancy progresses, leading to an inability of being able to satisfy the energy requirements for the jenny and the foal. Thus, it is a major predisposing factor for hyperlipemia in the late pregnancy stage of jennies [24]. In this study, we compared the levels of lipids, biochemical parameters, selected antioxidant elements and oxidative stress parameters for jennies with and without hyperlipemia. Our results provide a deep understanding of evaluating the features of hyperlipemia in jennies.

We observed serum lipid metabolism disorders that increased levels of TGs, T-CHO and LDL-C in jennies with hyperlipemia. Similar results were presented in the previous findings for donkeys and pony mares [23,25]. Serum TG is commonly used to determine the risk of a donkey becoming hyperlipemic. Donkeys with serum TG concentrations higher than the normal range should begin treatment to correct negative energy balance [26]. Hyperlipemia in donkeys may be due to an increased secretion of VLDLs by the liver, disturbed catabolism of VLDLs and decrease in TGs [27]. This increased LDL-C concentration in the serum was associated with the deficiency of the LDL-C receptor through its failure to function properly [6].

Oxidative stress plays a key role in the pathogenesis of chronic hyperlipemia. The accumulation of high levels of ROS induces oxidative stress, which leads to the damage of DNA, proteins and lipids. In the present study, the lower serum level of ALB and higher serum biochemical parameters related to liver function, including AST, ALT, AKP and CHE, were observed in hyperlipemic jennies. The serum concentration of ALB reflects a balance between ALB synthesis in the liver and its catabolism. ALB also functions as an extracellular antioxidant [28]. The findings of the elevation in the activity of AST, ALT, AKP and CHE in hyperlipemic jennies are in concordance with those reported for hyperlipemic rats [29]. This represents an increase in oxidative factors due to a high serum lipid concentration, which reduces antioxidant defenses and increases lipid peroxidation in the liver.

To date, little has been known about serum trace elements and vitamin change in hyperlipemia in jennies. Evaluating their potential necessity would be valuable. We found significantly lower serum levels of Se in hyperlipemic jennies compared to the control group. Nonetheless, the analysis of serum Se alterations in the published studies on humans with dyslipidemia is highly controversial [30]. Se is an essential trace element that has the ability to counteract free radicals and protect the structure and function of proteins, DNA and chromosomes from oxidative injury [31]. Its biological function is expressed through biologically active compounds, including glutathione peroxidases and other selenoproteins, which have important enzymatic functions associated with antioxidant activity. Furthermore, our results show that the serum level of Se positively correlates with TGs and ALB in hyperlipemic animals, similar to the results previously reported for humans [30,32]. Indeed, Se has been regarded as one of the important factors for lipid metabolism [33]. The evidence supports the idea that Se plays a vital role in insulin mimicry and anti-diabetes [34,35]. Donkeys have innate insulin resistance, especially obese or hyperlipemic jennies [6]. Our present findings indicate an impaired function of selenoproteins and liver metabolism, and the antioxidant capacity of the jennies may have been compromised during late pregnancy. The detailed biological process of selenoproteins involved in lipid metabolism in hyperlipemic jennies needs to be further investigated.

Zinc is known to contribute to the metabolic processes of lipids [17,36]. A previous study found a decrease in serum Zn levels in overweight and obese individuals [37]. However, our study shows no significant differences in serum Zn levels between the two groups. Consistent with our findings, a systematic review confirmed that serum zinc was not irrelevant to dyslipidemia in human studies [17]. The discrepant results may be due to different types of blood lipids and the high intersubject variability in the study population. In addition, our results show that the serum level of Zn is positively associated with the activity of T-AOC in jennies with hyperlipemia. Zn is an integral part of key antioxidant enzymes, and Zn deficiency impairs their synthesis, resulting in increased oxidative stress [38]. A systematic review and meta-analysis showed that Zn supplementation has favorable effects on plasma lipid parameters [39].

An antioxidant defense system, comprising enzymes such as T-SOD and non-enzymatic compounds (e.g., GSH), can prevent oxidative damage to lipoproteins in the blood [40]. In the present study, we observed decreased activities of serum T-SOD and GSH in jennies with hyperlipemia, resulting in a significantly limited antioxidant capacity. The significant decline in GSH may be due to enhanced oxidation or its consumption by electrophilic compounds, such as lipid peroxidation aldehydes [41]. Furthermore, it has been reported that there is a decreased GSH concentration in erythrocytes from hyperlipemic individuals, which has been found to be partly related to high cholesterol levels [42]. Additionally, oxidative stress may produce toxic substances, such as MDA. The formation of MDA is commonly considered a hallmark of cell damage and hepatic injury [40].

Based on the ROC analysis, the AUCs for T-CHO, ALB, AKP, CHE, T-SOD, GSH and MDA were relatively high (0.972, 0.889, 1.000, 0.951, 1.000, 0.917 and 1.000, respectively). These indices may be clinically useful with respect to screening for hyperlipemia in late pregnant jennies. However, serum selenium, zinc and vitamin E could not be used for evaluating the severity of donkey hyperlipemia due to the lower AUC.

## 5. Conclusions

In summary, significant alterations were observed in various serum lipids, biochemical parameters, antioxidant elements and oxidative stress parameters in jennies with and without hyperlipemia. These changes reflect the metabolic disorders, liver dysfunction and oxidative stress in late pregnant hyperlipemic jennies, providing a basis for the improvement of clinical diagnostic methods and the early prevention and control of hyperlipemia in jennies. The exact effect of antioxidant supplements, such as Se and enzymes, to prevent lipid disorders in late pregnant jennies should be verified in additional specifically designed clinical studies.

## Figures and Tables

**Table 1:** Comparison of age, parity, body weight, BCS and gestation length in hyperlipemic (*n* = 20) and healthy (*n* = 20) jennies.

Parameters	Hyperlipemia	Healthy	*p* Value
Age	6.1± 0.2	5.8 ± 0.2	0.403
Parity	1.9 ± 0.1	1.8 ± 0.2	0.687
Body weight (kg)	209.4 ± 4.1 ^b^	247.5 ± 3.8 ^a^	0.000
BCS	1.9 ± 0.1 ^b^	3.1 ± 0.1 ^a^	0.000
Gestation length (days)	367.2 ± 1.6	362.7 ± 1.5	0.392

Abbreviations: BCS, body condition score. ^a,b^ In the same row, values with different letters in superscript differ significantly (*p* < 0.05).

**Table 2:** Comparison of serum lipids for hyperlipemic (*n* = 20) and healthy (*n* = 20) jennies.

Parameters		Hyperlipemia	Healthy	*p* Value
TG (mmol/L)	Mean ± SE	21.35 ± 1.44 ^a^	1.84 ± 0.14 ^b^	0.000
	95% CI	18.31~24.39	1.55~2.12	
T-CHO (mmol/L)	Mean ± SE	10.75 ± 1.14 ^a^	3.50 ± 0.15 ^b^	0.000
	95% CI	8.36~13.15	3.20~3.79	
HDL-C (mmol/L)	Mean ± SE	1.85 ± 0.22 ^b^	2.86 ± 0.16 ^a^	0.002
	95% CI	1.37~2.32	2.53~3.18	
LDL-C (mmol/L)	Mean ± SE	3.46 ± 0.37 ^a^	1.96 ± 0.15 ^b^	0.001
	95% CI	2.69~4.23	1.66~2.26	

Abbreviations: TG, triglyceride; T-CHO, total cholesterol; HDL-C, high-density lipoprotein cholesterol; LDL-C, low-density lipoprotein cholesterol; SE, standard error; CI: confidence interval. ^a,b^ In the same row, values with different letters in superscript differ significantly (*p* < 0.05).

**Table 3:** Comparison of serum biochemical parameters related to liver function between hyperlipemic (*n* = 20) and healthy (*n* = 20) jennies.

Parameters		Hyperlipemia	Healthy	*p* Value
TP (g/L)	Mean ± SE	77.80 ± 2.79	75.55 ± 1.61	0.490
	95% CI	71.97~83.64	72.16~78.94	
ALB (g/L)	Mean ± SE	25.84 ± 0.96 ^b^	31.98 ± 1.22 ^a^	0.001
	95% CI	23.72~27.96	29.29~34.68	
AST (U/L)	Mean ± SE	68.51 ± 20.41 ^a^	37.33 ± 3.87 ^b^	0.019
	95% CI	21.45~115.56	29.44~45.21	
ALT (U/L)	Mean ± SE	22.41 ± 3.24 ^a^	13.08 ± 1.21 ^b^	0.013
	95% CI	15.61~29.21	10.65~15.51	
AKP (King unit/100 mL)	Mean ± SE	66.70 ± 6.45 ^a^	15.08 ± 0.73 ^b^	0.000
	95% CI	53.20~80.20	13.55~16.61	
CHE (U/mL)	Mean ± SE	4.15 ± 0.19 ^a^	2.11 ± 0.19 ^b^	0.000
	95% CI	3.75~4.55	1.71~2.52	

Abbreviations: TP, total protein; ALB, albumin; AST, aspartate aminotransferase; ALT, alanine aminotransferase; AKP, alkaline phosphatase; CHE, cholinesterase; SE, standard error; CI: confidence interval. ^a,b^ In the same row, values with different letters in superscript differ significantly (*p* < 0.05).

**Table 4:** Comparison of serum selenium, zinc and vitamin E in hyperlipemic (*n* = 20) and healthy (*n* = 20) jennies.

Parameters		Hyperlipemia	Healthy	*p* Value
Se (μg/L)	Mean ± SE	42.19 ± 4.65 ^b^	55.51 ± 3.12 ^a^	0.031
	95% CI	31.67~52.71	48.32~62.71	
Zn (mg/L)	Mean ± SE	1.60 ± 0.13	1.45 ± 0.09	0.345
	95% CI	1.29~1.90	1.24~1.65	
VE (nmol/L)	Mean ± SE	560.80 ± 43.07	552.58 ± 43.33	0.894
	95% CI	463.36~658.24	454.55~650.61	

Abbreviations: Zn, zinc; Se, selenium; VE, vitamin E; SE, standard error; CI: confidence interval. ^a,b^ In the same row, values with different letters in superscript differ significantly (*p* < 0.05).

**Table 5:** Comparison of serum oxidative stress parameters for hyperlipemic (*n* = 20) and healthy (*n* = 20) jennies.

Parameters		Hyperlipemia	Healthy	*p* Value
T-AOC (mmol/L)	Mean ± SE	0.67 ± 0.01 ^b^	0.71 ± 0.01 ^a^	0.004
	95% CI	0.65~0.68	0.69~0.72	
GSH (μmol/L)	Mean ± SE	2.87 ± 0.35 ^b^	9.92 ± 1.06 ^a^	0.000
	95% CI	2.15~3.59	7.70~12.13	
T-SOD (U/mL)	Mean ± SE	70.80 ± 0.15 ^b^	76.85 ± 0.18 ^a^	0.000
	95% CI	70.49~71.12	76.47~77.23	
MDA (nmol/mL)	Mean ± SE	6.34 ± 0.47 ^a^	2.78 ± 0.09 ^b^	0.000
	95% CI	5.36~7.32	2.59~2.96	

Abbreviations: T-AOC, total antioxidant capacity; GSH, glutathione; T-SOD, total superoxide dismutase; MDA, malondialdehyde; SE, standard error; CI: confidence interval. ^a,b^ In the same row, values with different letters in superscript differ significantly (*p* < 0.05).

## Data Availability

The data presented in this study are available on request from the corresponding authors.
